# Normative data in resting and maximum heart rates and a prediction equation for young Tunisian soccer players: a cross-sectional study

**DOI:** 10.17179/excli2023-6215

**Published:** 2023-07-17

**Authors:** Hatem Ghouili, Zouhaier Farhani, Sofiane Amara, Soukaina Hattabi, Amel Dridi, Noomen Guelmami, Anissa Bouassida, Nicola Bragazzi, Ismail Dergaa

**Affiliations:** 1 Research Unit, Sportive Performance and Physical Rehabilitation, High Institute of Sports and Physical Education of Kef, University of Jendouba, Kef, Tunisia; 2 Research Unit (UR17JS01) Sports Performance, Health & Society, Higher Institute of Sport and Physical Education of Ksar Saîd, Universite de la Manouba, Tunis, Tunisia; 3 Postgraduate School of Public Health, Department of Health Sciences (DISSAL), University of Genoa, Genoa, Italy; 4 Laboratory for Industrial and Applied Mathematics (LIAM), York University, Canada; 5 Primary Health Care Corporation (PHCC), Doha, Qatar

**Keywords:** maximum heart rate, resting heart rate, prediction equation, percentile curves, soccer players

## Abstract

Heart rate (HR) is an important indicator of work intensity during physical activity. Maximum heart rate (MHR) is a physiological measure that is frequently used as a benchmark for maximal exercise intensity. The aim of this study was to establish reference curves for maximum heart rate (MHR) and resting heart rate (RHR) and to develop an estimated equation for Tunisian adolescent footballers. The study involved 801 adolescent players, aged 11 to 18, who belonged to five Tunisian first-division soccer teams. The LMS method was used for smoothing the curves and the multivariate linear regression to develop a prediction equation of MHR. Our results showed that MHR and RHR reference curves decrease with age. The values of the median curves of MHR and RHR ranged from 208.64 bpm (11 years) to 196.93 (18 years) and 73.86 (11 years) to 63.64 (18 years), respectively. The prediction equation obtained from the model was MHR= 225.08 - 1.55 X Age (years) (R^2 ^= 0.317; P < 0.001; standard error of the estimate (SEE) = 5.22). The comparisons between the estimated values and the measured values have found that our model (- 0.004 ±5.22 bpm) was to be more accurate than two other widely known models. BOX's equation underestimates the measured MHR values by -3.17 ± 5.37 bpm and TANAKA's equation overestimates by + 4.33 ±5.5 bpm. The reference curves can be used by coaches and physical trainers to classify the resting heart rate (RHR) and maximum heart rate (MHR) of each adolescent player, track their evolution over time, and design tailored training programs with specific intensities for Tunisian soccer players.

## Introduction

The maximum heart rate (MHR) is the highest heart rate achieved during a graded exercise performed at maximum effort, and it can be measured in the laboratory or in the field. MHR determines the upper limit of cardiovascular function (Mahon et al., 2010[18]). MHR is a physiological measure that is frequently used as a benchmark for maximal exercise intensity to avoid errors when defining training loads (Antonacci et al., 2007[3]). This is owing to the established linear relationship between heart rate (Zanuto et al., 2020[31]) and oxygen consumption (VO_2_) in activities with progressive intensity (da Silva et al., 2021[7]). According to this relationship, percent of MHR corresponds to a percentage of VO_2_max at a particular effort intensity. Therefore, we can supervise the HR in % MHR and control the intensity of a given physical activity, thus personalized the physical training (Antonacci et al., 2007[3]).

Coaches and physical trainers in soccer frequently base training HR intensities on MHR, and that in cases where direct measurement of MHR is not possible, an equation based on age can be used to predict MHR. FOX's equation (MHR = 220 - age) (Fox et al., 1971[10]) and TANAKA's equation (MHR= 208 - 0.7 x age) (Tanaka et al., 2001[29]) are two widely used equations in sports practices. The validity of these equations has been rigorously tested in large samples of adults who are healthy, sedentary, athletes and persons with health complications. In contrast, few investigations in children and adolescents have been conducted (Nikolaidis, 2015[20]). In a given population, MHR values demonstrate a large inter-individual variation. This may make estimated MHR using linear equations more inaccurate. It is strongly influenced by a person's physiology and lifestyle (Sarzynski et al., 2013[28]).

Reference percentile curves are widely used in medical practice as a screening tool during the important period of childhood. They identify abnormal subjects, in the sense that their value of a given measure is located in one or the other tail of the reference distribution (Borghi et al., 2006[4]). An important aspect of this process is drawing the curves in percentiles, which are generally presented as the frequency distribution of values based on gender and age. The graphs' common pattern is shown as a series of smooth curves drawn through the points of each age and transformed into a normal distribution (Cole, 2012[6]; Ghouili et al., 2018[12]). 

During the last decades, several countries have established their references or models of physical and physiological parameters in the fields of health and physical activity of trained or untrained subjects (Eytan et al., 2017[9]; Lesinski et al., 2020[15]; Santos et al., 2014[27]). For example, a recent study showed that athletes practicing endurance sports have similar MHR values as those engaged in speed and strength activities, and both have lower values than untrained subjects. This highlights the need to review popular prediction equations in sport fields (Nikolaidis, 2015[20]). Therefore, the objective of the present study is to develop smoothed reference percentile curves of MHR and resting heart rate (RHR) based on age (11-18 years), as well as a predicted equation specifically for Tunisian young soccer players. 

## Methods

### Data collection and procedure

In the pre-season of 2022/2023, we collected a cross-sectional data from 801 football players aged 11 to 18. The subjects are males and were recruited from nine teams in national first division in Tunisia. The participants had regularly participated in at least three training sessions per week and in competitions recognized by the Tunisian Football Federation. 

The players voluntarily participated, and they have possibility of leaving the study at any time without justification. They informed their parents of the methods and objectives used to assess their abilities.

In accordance with the Declaration of Helsinki (2013), the research has been fully approved by the Ethics Committee of the Higher Institute of Physical Education and Sports in Kef (22-2023).

### Anthropometric parameters

Height, body mass and skinfolds were measured using precise tools, including electronic scale to the nearest 0.1 kg (HD-351; Tanita, Arlington Heights, Illinois, USA), portable stadiometer to the nearest 0.001 m (SECA Leicester, United Kingdom) and caliper to the nearest 0.5 mm (Harpenden, West Sussex, UK).

Body mass index was calculated by the ratio of body mass (kg) to square height (m²), and body fat (BF) was estimated from the sum of 4 skinfolds (Triceps, Biceps, Subscapular Suprailiac) using Durnin and Womersley (1974[8]) formula;

**BF % = (4.95 / D) - 4.50**

where BF is the estimated body fat percentage and D is the density of the body, which is calculated using the sum of the four skinfolds (in millimeters) according to the equation:

**D = 1.0994921 - (0.0009929 x sum of skinfolds) + (0.0000023 x sum of skinfolds²) - (0.0001392 x age)**

### Graded exercise test

The participants performed a running test between two lines separated by 20 meters following the signal of an emitted audio. The initial speed was 8.5 km/h and increased by 0.5 km/h at each one-minute stage (Léger et al., 1988[14]). Heart rate (HR) was continuously monitored using Polar Team Sport System (Polar-Electro OY, Kempele, Finland). The test ended when the participant could not reach the beep line by two consecutive laps or wished to stop the test.

### Smoothed reference curves

The lambda, mu and sigma (LMS) method describes the distribution of the different measurements in the reference percentile curves by three curves: the median, the coefficient of variation and the asymmetry curves (Cole, 1990[6]).

The percentile curves were developed using the following equation:

**C** **_100α_** **= M(1 + LSz** **_α_** **)^1/L**

### Statistical analysis

Data analyses were performed using the Statistical Package for the Social Sciences (SPSS, version 28.0). Data are presented as means and standard deviations (SD) for all measured variables. Relationships between MHR and continuous variables (weight, height, BMI, body fat percentage, RHR) were provided using Pearson's correlation coefficient. One-way analysis of variance (ANOVA) and subsequent Bonferroni post-hoc test (if differences between groups were revealed) were used to examine differences between successive age group values. In addition, the stepwise method of multivariate linear regression was used to identify predictors of MHR after checking the regression hypotheses (homoscedasticity, multicollinearity, and normal distribution of residuals). Root Mean Squared Error (RMSE) was used to assess the goodness-of-fit of the model. Examination of the accuracy and the variability of the prediction equations was performed by the analysis of Bland, Altman graphical method (Altman and Bland, 1983[2]). 

## Results

The descriptive characteristics of the sample are presented in Table 1(Tab. 1).

The sample was distributed over the eight age groups as follows: 12.23 % (11 years), 10.61 % (12 years), 13.61 % (13 years), 13.48 % (14 years), 13.11 % (15 years), 12.86 % (16 years), 10.99 % (17 years) and 13.11 % (18 years). There were significant differences between all successive age groups in terms of height, body mass, and trainability. However, the differences were particularly noticeable for a certain age group in other variables. For BF %, there were significant differences between the 11/12 years (p<0.001), 13/14 years (p<0.0001), and 16/17 years (p<0.01) age groups. For MHR, there were significant differences between the 11/12 years (p<0.001), 14/15 Years (p<0.01), and 17/18 years (p<0.001) age groups. For RHR, there was a significant difference only between the 11/12 years (p<0.001) age group (Table 1(Tab. 1)). 

### Reference values

**Figure 1:**
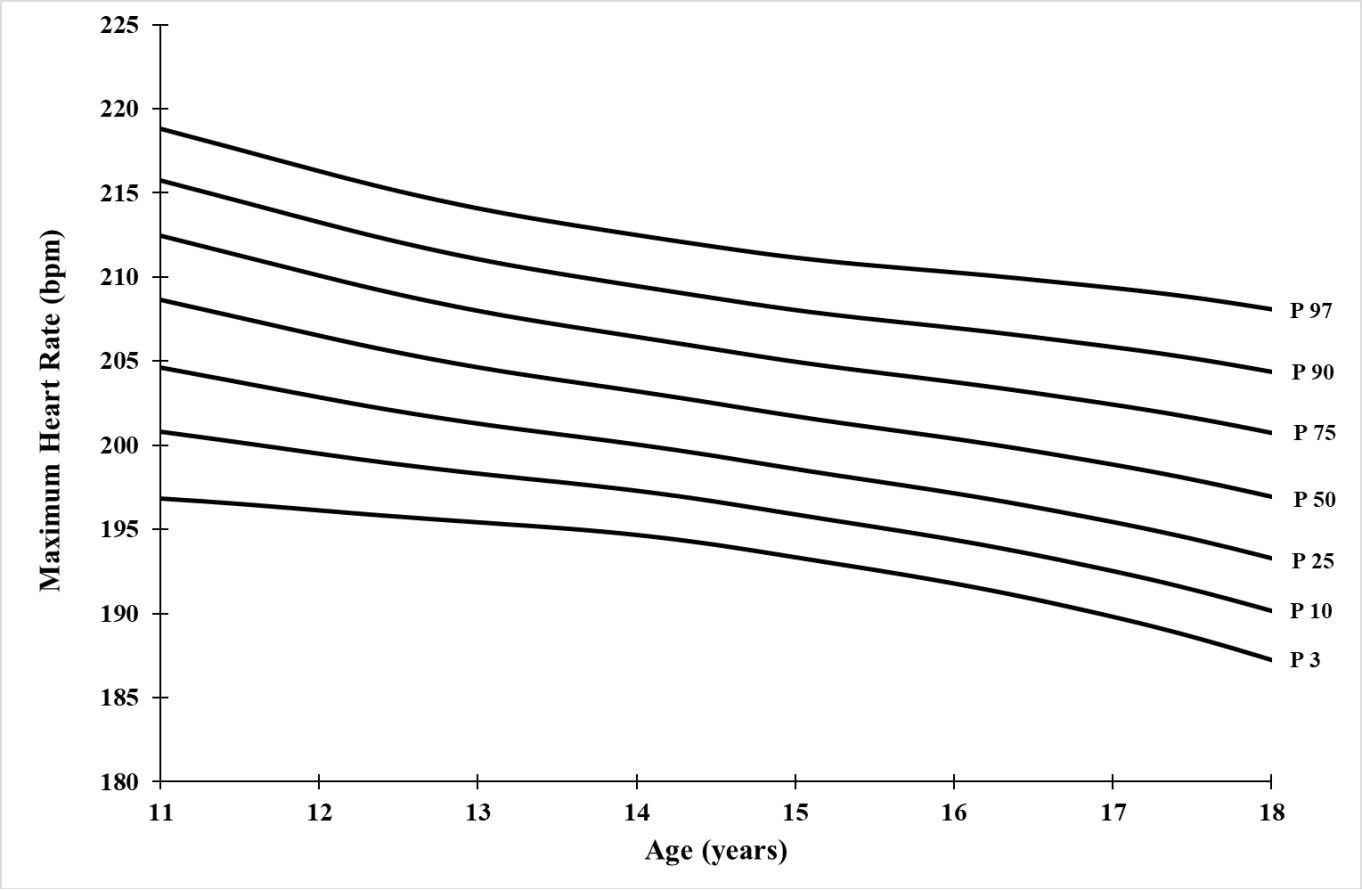
Smoothed percentiles curves of Maximum Heart rate (MHR) for Tunisian soccer players aged 11-18 years

**Figure 2:**
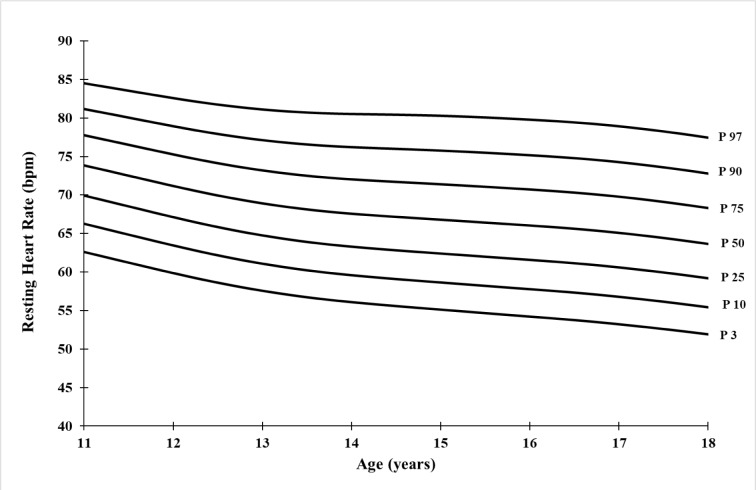
Smoothed percentiles curves of Resting Heart rate (RHR) for Tunisian soccer players aged 11-18 years

The median curve values for MHR and RHR vary from 208.64 bpm (11 years) to 196.93 bpm (18 years) and from 73.86 bpm (11 years) to 63.64 bpm (18 years), respectively. The lower percentile values (P3) decrease from 196.83 bpm (11 years) to 187.23 bpm (18 years) for MHR, and from 62.59 bpm (11 years) to 51.94 bpm (18 years) for RHR. Similarly, the upper percentile values (P97) decrease from 218.81 bpm (11 years) to 208.10 bpm (18 years) for MHR, and from 84.52 bpm (11 years) to 77.44 bpm (18 years) for RHR (Table 2(Tab. 2), and Figures 1(Fig. 1) and 2(Fig. 2)).

The Pearson correlation coefficients between the MHR and the different variables (age, trainability, Body mass, height, BMI, Fat mass percent, RHR) are presented in Table 3(Tab. 3). All the variables had a strong negative correlation with the MHR.

### MHR prediction equations

Multivariate linear regression identified two models for production MHR. The first model included age as the only significant predictor using the following prediction equation:

**MHR= 225.08 – 1.55 X Age (years) **(1)

(R^2^ = 0.317, P <, 0.001, standard error of the estimate (SEE) = 5.22).

The error range for this model was -22.47 to +13.66 bpm. The mean difference between the measured and predicted MHR using this equation was -0.004 with a standard deviation of 5.22 bpm.

The second model revealed that both age and BMI were significant predictors of MHR

**MHR= 228.03 – 1.45 x Age (years) – 0,21x BMI (kg/m²) **(2)

(R^2^ = 0.324, P < 0.001, standard error of the estimate (SEE) = 5.20.

The error range of second model was -21.95 to +14.62 bpm. The mean difference was -0.066 with a standard deviation of 5.19 bpm.

The two developed models had similar percentages of estimated variation in MHR (31.7 % vs. 32.4 %) and SEE values (5.22 vs. 5.20 bpm). As result, we chose the first equation as the predictor of MHR values for young Tunisian soccer players because it only relied on one variable, which was age.

**Figure 3:**
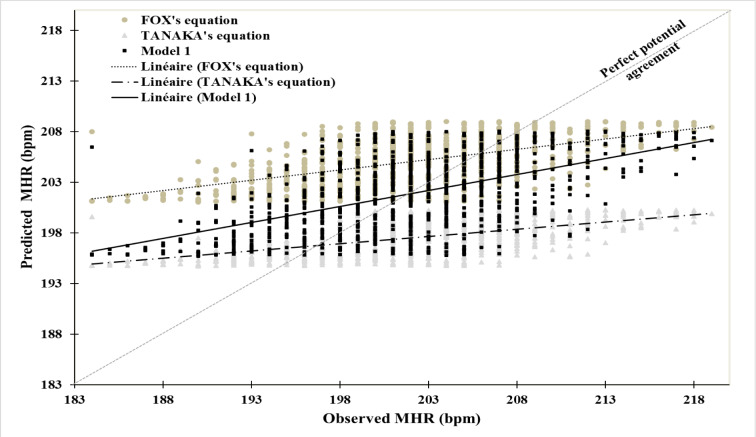
Potential agreement between observed and predicted values using three different equations, including our new prediction equation 1 (Model 1), BOX’s equation and TANAKA’s equation

Figure 3(Fig. 3) illustrate the potential agreement between the observed MHR and the predicted MHRs using three equations. Our model 1 demonstrated a stronger linear trend closer to the ideal trend than both FOX's and TANAKA's equations.

**Figure 4:**
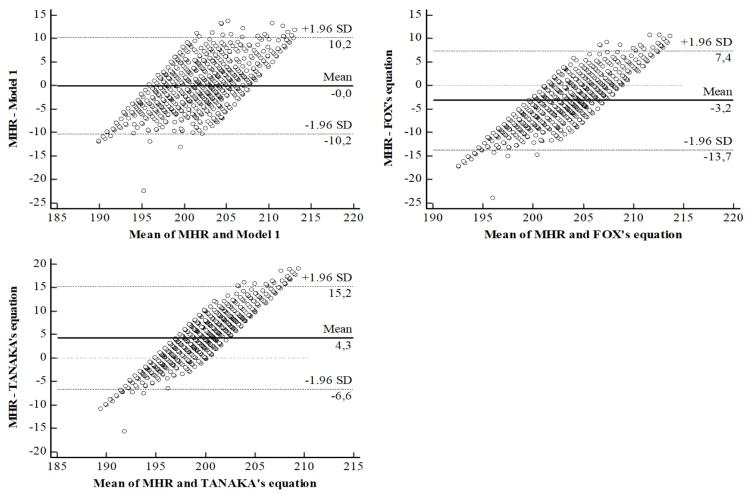
Bland Altman plots of the difference between observed and predicted values using three different equations: Model 1, BOX’s equation and TANAKA’s equation

Bland and Altman graph shows the high degree of agreement between our model 1 and the measured MHR values. Our model 1 demonstrated near-zero bias (+0.003) and upper and lower limits of agreement of +10.23 bpm and -10.24 bpm, respectively (see Figure 4(Fig. 4)).

For the FOX’s and TANAKA’s equations, the Bland and Atman graph illustrated that the biases are not negligible, with values of -3.17 bpm and +4.33 bpm, respectively. The upper and lower limits of agreement for the FOX’s equation are -7.35/+13.70 bpm, and for the TANAKA’s equation they are -15.25/+6.60 bpm (see Figure 4(Fig. 4)).

See also the Supplementary data.

## Discussion

The aim of this study was to develop reference curves of MHR and RHR and produce an estimated equation for children and adolescents' soccer players.

The 3^rd^, 50^th^, and 95^th ^percentile values of MHR showed a continuous decrease with age, through variation of decline of around 1.67 ± 0.30 bpm, 1.37 ± 0.72 bpm, and 1.53 ± 0.62 bpm per year, respectively, resulting in an overall decline rate of -4.88 %, -5.61 %, and -4.89 % between the ages of 11 and 18 years. Tanaka et al. (2001[29]) have demonstrated that the decline in MHR is not related to physical activity level or gender, but rather primarily due to the reduction in intrinsic heart rate. A constant relationship between the variation of heart rate and the intrinsic contractile function of the myocardium has been found in previous studies by Jose and Taylor (1969[13]) and Opthof (2000[22]).

Smoothed percentile values for RHR are also inversely associated with age. Indeed, the values for the 3^rd^, 50^th^, and 97^th^ percentiles vary by 1.52 ± 0.72 bpm, 1.46 ± 0.75 bpm, and 1.01 ± 0.62 bpm per year, respectively, with a total percentage decrease of -17.01 %, -13.84 %, and -8.38 % from 11 to 18 years of age. In the same context, Zanuto et al. (2020[31]) found, through simple regression analysis, that RHR was associated with age (β = -1.61; 95 % CI: -2.11, -1.16) and total physical activity (β = -0.51; 95 % CI: -0.91, -0.11) parameters in boys aged 10 to 17 years.

In a meta-analysis review, Pieles and Stuart (2020[25]) showed that training-related changes in adolescent athletes include increased cardiac output, as well as increased size and thickness of the ventricular cavity, with a decreased RHR. Training three days per week for 12 weeks with high-intensity physical activity for 15 minutes each day resulted in significant improvements in systolic blood pressure and RHR in school children.

Zanuto et al. (2020[31]) described that the decrease in resting heart rate under the effects of physical activity may provide three action mechanisms: (1) neural mechanism, which regulates the necessary stimuli through the action of muscle afferent nerves (chemoreceptors and mechanoreceptors) based on exercise intensity, (2) baroreflex mechanism, which acts as a behavioral regulator with every beat, and (3) central mechanism, which controls changes in sympathetic and parasympathetic efferent activity during exercise (Zanuto et al., 2020[31]). In other words, moderate to vigorous physical activity promotes health benefits by producing physiological changes in the cardiovascular, pulmonary, and metabolic systems (Nystoriak and Bhatnagar, 2018[21]).

Several variables, including anthropometric parameters, age, trainability, and RHR variables, were examined to identify the best predictor of MHR. Our analysis revealed that age is the most significant predictor, with an inverse correlation of -0.56 (p<0.001) with MHR. Previous studies have shown varying degrees of correlation between MHR and age across different age groups of athletes. For example, a correlation coefficient of -0.41 (p<0.001) was reported for soccer players aged 11-36 years (Nikolaidis, 2015[20]), while a weaker correlation (-0.28; p<0.001) was found for athletes aged 9 to 18. In contrast, studies covering larger age ranges reported much stronger correlations, such as -0.60 for age groups of 14-77 years (Whaley et al., 1992[30]) and 19-89 years (Nes et al., 2013[19]). In our study, we developed two new prediction equations for MHR in young Tunisian soccer players, based on age and BMI as independent variables. The first model, which uses only age as a predictor, had R² of 0.317 with SEE of 5.22. The second model, which includes both age and BMI, had an R² of 0.324 with SEE of 5.20. Since the two models had similar R² values and SEE, we chose the simpler model that includes only age as the predictor for MHR.

After analyzing the difference between observed MHR values of young Tunisian soccer players and the predicted values using our model 1, as well as the BOX's equation and TANAKA's equation, we observed biases and standard deviations of -0.003 ± 5.22, -3.17 ± 5.37, and +4.33 ± 5.57, respectively. The errors that exceed the limits of agreement can reach up to 3.6 % for our model 1, 4.8 % for the BOX's equation, and 5.4 % for TANAKA's equation. Our findings align with previous research by Gellish et al. (2007[11]) and Pereira Rodriguez et al. (2016[24]), indicating that the BOX's equation underestimates MHR values, while TANAKA's equation overestimates them. However, some studies, such as those by Nikolaidis (2015[20]) and Papadopoulou et al. (2019[23]), have reported conflicting results, showing that BOX's equation overestimates and TANAKA's equation underestimates MHR in adolescents. According to Almeida et al. (2010[1]), predictive equations become valid when they are applied to populations with similar characteristics to those of the sample for which the equation was developed (Machado and Denadai, 2011[16]). TANAKA's prediction equation was developed using a meta-analysis approach that extracted MHR values from 18,712 healthy adult subjects of 492 different groups in 351 studies (Tanaka et al., 2001[29]). The BOX model was based on an estimate derived from the observation of a linear best fit of a series of raw and averaged data compiled in 1971 (Fox et al., 1971[10]), and research since 1971 has revealed errors in the estimation of MHR, rendering the BOX's prediction equation inaccurate (Robergs and Landwehr, 2002[26]).

Numerous authors have attempted to develop alternative formulas for predicting MHR, but the resulting equations may not be applicable to the general population due to various factors, such as the sample size, age group, and type of stress test (Magrì et al., 2022[17]).

Our study has several strengths. (1) We developed reference values for MHR and RHR that allow for ranking players according to their aerobic capacity compared to those of subjects of the same age and from the same population. (2) We included several parameters, such as age, stature, body mass, BMI, percentage of body fat, trainability, and RHR, as possible predictors for MHR in young Tunisian footballers. (3) We developed first prediction equation for MHR during the adolescent period. However, our study also has some limitations. For instance, the equation we developed only has a 30 % predictive capacity and is applicable to a little age group of 11-18 years. Therefore, future studies should expand the age group by combining the period of adolescence with that of young adult footballers. Additionally, longitudinal studies would be beneficial to better specify the tendencies of the curves of references over time.

## Conclusion

Based on these results, it is suggested that the reference curves developed from the Tunisian children and adolescent sample can be used to classify RHR and MHR of each player and to monitor their changes over time. In the context of testing and training, practitioners are advised to use the prediction equation developed in this study, which is more accurate than BOX's and TANAKA's equations. Finally, the reliability of the tools developed in this study needs to be tested by Tunisian coaches and physical trainers.

## Supplementary Material

Supplementary data

## Figures and Tables

**Table 1:**
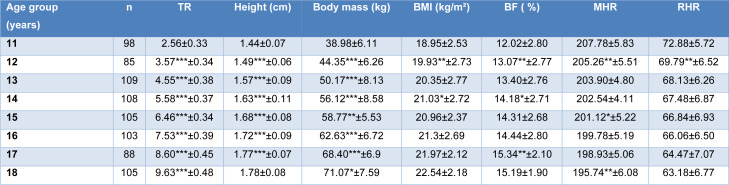
Characteristics of anthropometric measurements (height, body mass, body mass index (BMI), body fat (BF)), trainability (TR), maximum heart rate (MHR), resting heart rate (RHR) by age group of study participants *: P< 0.01; **: P< 0.001; ***: P< 0.0001; differences between successive age group values

**Table 2:**
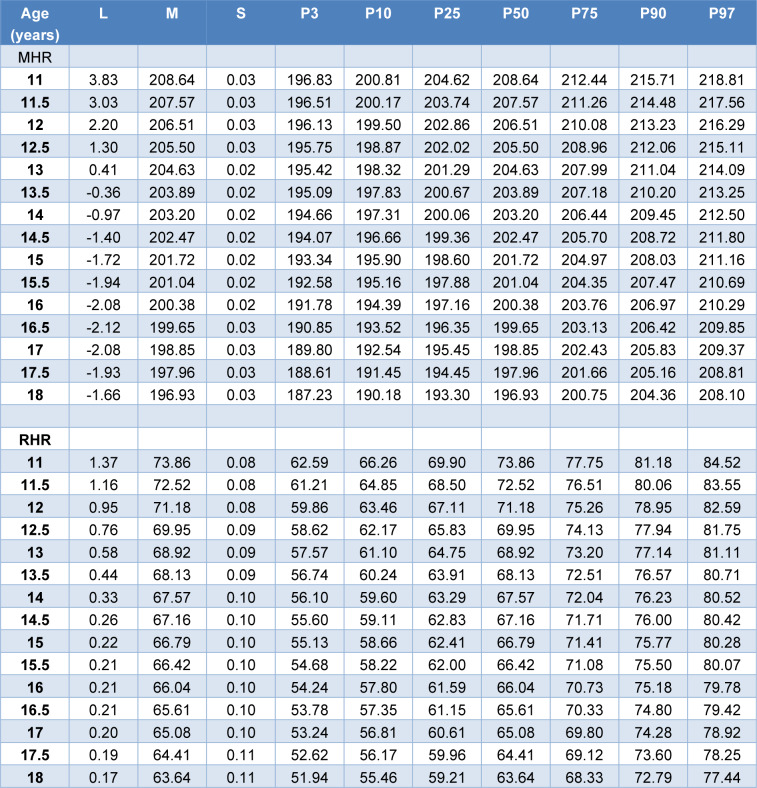
Lambda (L), Median (M), Coefficient of Variation (S) and Percentile curves of Maximum Heart rate (MHR) (bpm) and Resting Heart rate (RHR) (bpm) for Tunisian soccer players aged 11-18 year

**Table 3:**
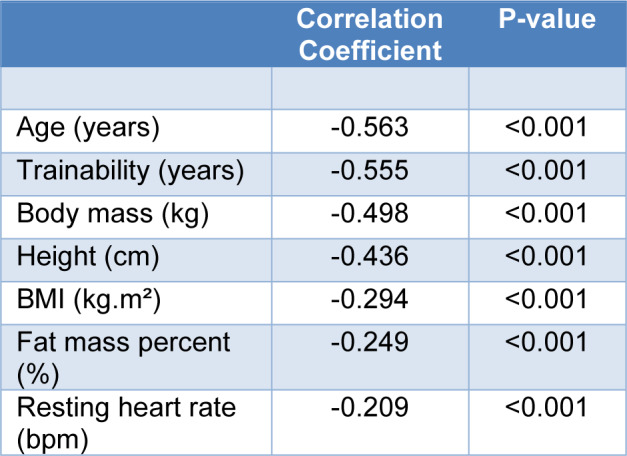
Pearson Correlation Coefficients Between Observed Maximal Heart Rate (bpm) and the independents variables.
